# Double-diabetes in a real-world sample of 2,711 individuals: associated with insulin treatment or part of the heterogeneity of type 1 diabetes?

**DOI:** 10.1186/1758-5996-7-S1-A129

**Published:** 2015-11-11

**Authors:** Fernando de Mello Almada Giuffrida, Caroline Bulcão, Roberta A Cobas, Carlos Antonio Negrato, Marilia B Gomes, Sergio Atala Dib

**Affiliations:** 1 CEDEBA, Salvador, Brazil

## Background

Double diabetes (DD) can describe both individuals with obesity upon diagnosis of type 1 diabetes (T1D) and those who have gained weight during follow up, although most of the first group has been excluded from classic T1D trials. The interrelation between weight excess and cardiovascular risk factors (CVRF) is not well understood in these individuals.

## Objective

To evaluate the frequency of DD in a real-world T1D sample and the interaction of insulin treatment, diabetes duration, weight excess, and CVRF.

## Materials and methods

2,711 individuals with clinical diagnosis of type 1 diabetes from secondary diabetes care centers in 20 Brazilian cities, studied by the Brazilian Type 1 Diabetes Study Group (BrazDiab1SG), have been assessed regarding frequency of obesity according to diabetes duration, insulin dose per body weight according to diabetes duration and BMI status, CVRF according to diabetes duration and BMI.

## Results

Patients with diabetes duration < 5 and ≥ 5 yrs. had similar frequency of overweight (20.4 vs 25%) and obesity, (9.8% vs. 6.1%), p 0.28 for trend. Age according to BMI status was different among normal weight (N), overweight (OW), and obese individuals (Ob) in both diabetes duration < 5 yrs. (13.5±6.6 vs. 13.1±8.0 vs. 9.8±6.6 yrs. old, respectively, p<0.001) and diabetes duration ≥ 5 yrs. (24.2±11.5 vs. 26.3±12 vs. 25.6±13.9, respectively, p=0.003). HbA1c (%) was similar in all groups regardless of diabetes duration and BMI status. Insulin dose (U/kg/day) was lower in Ob individuals compared to N, with mean (95% CI) 0.72 (0.62-0.83) vs. 0.88 (0.84-0.92) U/kg/day for diabetes duration < 5 yrs. and 0.84 (0.77-0.92) vs. 0.99 (0.97-1.01) U/kg/day for duration ≥ 5 yrs. Ob individuals had lower HDL (47.5 [43.0-51.9] vs. 54.4 [53.0-55.8] mg/dL) and higher non-HDL-cholesterol (134.5 [123.2-145.9] vs. 115.2 [111.6-118.9] mg/dL) than N only among those with more than 5 yrs. of diabetes. Number of insulin applications was not associated with weight excess or diabetes duration.

## Conclusions

Lower insulin doses in obese individuals point to a role of clinical heterogeneity of insulin deficiency and sensitivity on the progression of DD. Lower levels of HDL-cholesterol and high number of cardiovascular risk factors are associated with obesity in long duration T1D. These data suggest a broad clinical landscape of pathophysiological phenomena for double diabetes, rather than simple progression of a homogeneous clinical entity.

